# Epidermoid cyst with unusual magnetic resonance characteristics and spinal extension

**DOI:** 10.1186/s12957-015-0651-1

**Published:** 2015-08-07

**Authors:** Jaejoon Lim, Kyunggi Cho

**Affiliations:** Department of Neurosurgery, Bundang CHA Medical Center, CHA University College of Medicine, Yatap-dong 59, Seongnam, 463-712 South Korea

**Keywords:** Epidermoid cyst, Magnetic resonance image, Cervical spine extension

## Abstract

Intracranial epidermoid cysts are generally located in the cerebellopontine and parasellar areas and appear hypo-dense on computed tomography and hypo-intense on T1-weighted magnetic resonance imaging. We report a case of an unusual epidermoid cyst of the cerebellopontine angle extending into the upper cervical canal that appeared hyper-dense on computed tomography scanning, hyper-intense on T1-weighted magnetic resonance (MR) images, and hypo-intense on T2-weighted MR images.

## Background

Epidermoid tumors represent remnants of ectodermal tissues misplaced during embryogenesis and account for approximately 0.3 to 1.8 % of intracranial tumors. They are most common in the cerebellopontine angle and suprasellar and parasellar regions [1, 2]. Intracranial epidermoid cysts generally appear as well-defined lobulated hypo-dense masses on computed tomography (CT) scan. On magnetic resonance (MR) studies, they typically appear hypo-intense on T1-weighted and hyper-intense on T2-weighted images [3, 4]. Occasionally, they may appear hyper-dense on CT scans and hyper-intense on T1-weighted MR images [5–8]. We report a case of epidermoid cyst which showed hyper-intensity on T1-weighted MR images with upper cervical extension.

## Case presentation

**Fig. 1:**
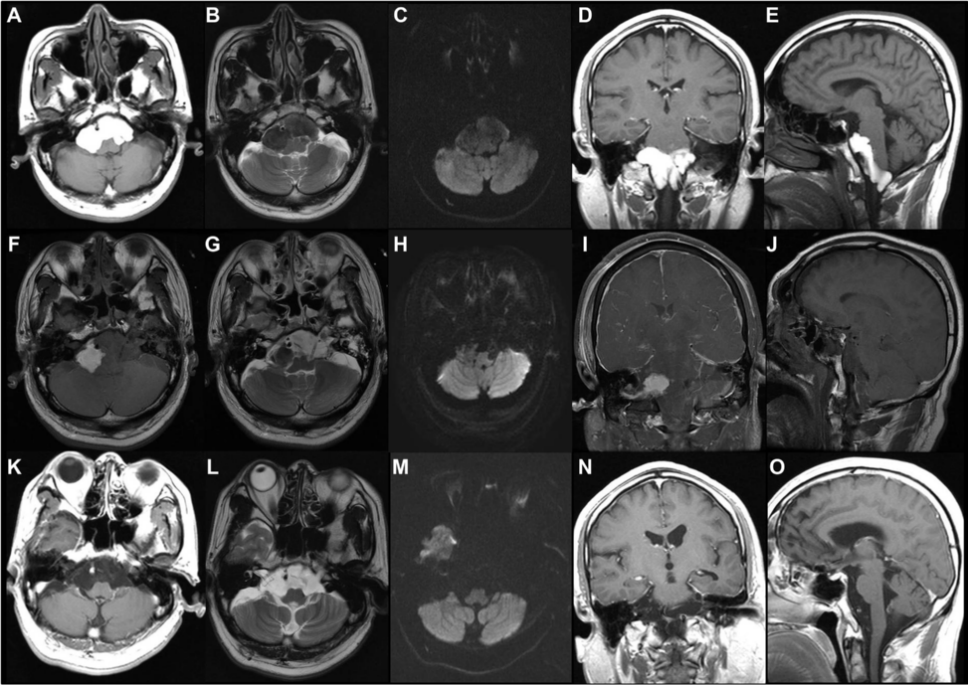
Preoperative and postoperative MR imaging. In the preoperative MR image, a well-defined, lobulated mass with a size of about 5.6 × 2.7 × 6.1 cm is seen in front of the pons to the medulla with several internal septae. The mass showed hyper-intensity on T1-weighted, hypo-intensity on T2-weighted, and hypo-intensity on diffusion-weighted images. The superior portion of this cystic lesion compresses the lower portion of the pons. The inferior portion of the lesion extends posteriorly and is surrounding the medulla, extending to the C1-C2 intervertebral level (**a**–**e**). Postoperative MR imaging after a grossly subtotal surgical resection was done through the minimal lateral supraorbital approach (**f**–**j**). In the 15-month follow-up MR images, the remnant cyst mass completely disappeared (**k**–**o**)

**Fig. 2:**
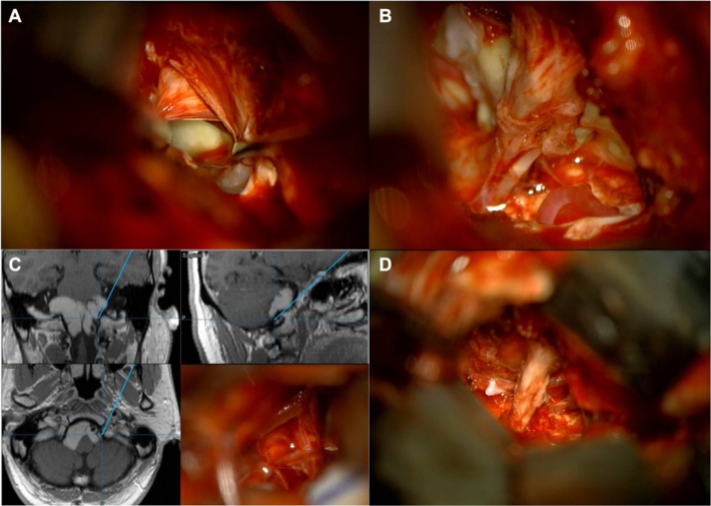
Operation images. **a** The trigeminal nerve (V2) and the petrous bone were exposed. **b** ,**c** The tumor in the anterior cerebellopontine area was seen. **d** The tumor was subtotally removed

**Fig. 3:**
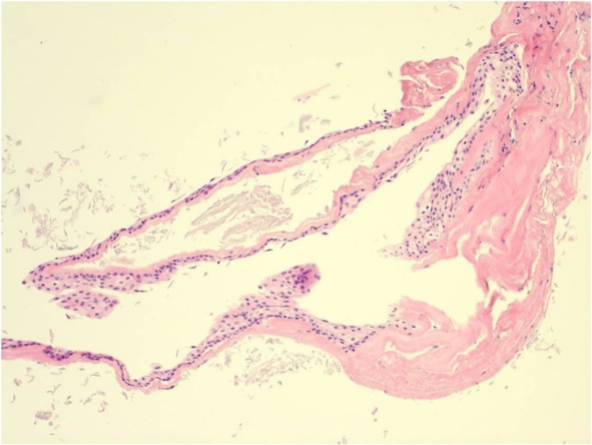
Pathologic examination. The cysts were thin-walled and lined by stratified squamous epithelium. Cystic contents included debris and keratin materials (H&E, ×100)

A 46-year-old man presented with tingling sensation and numbness on both lower extremities. CT showed hyper-dense masses in both cerebellopontine areas. In the preoperative MR image, a well-defined, lobulated mass with a size of about 5.6 × 2.7 × 6.1 cm is seen in the pons to the medulla area with several internal septae. The mass showed hyper-intensity on T1-weighted, hypo-intensity on T2-weighted, and hypo-intensity on diffusion-weighted images. This lesion shows no gadolinium contrast enhancement. The superior portion of this cystic lesion compressed the lower portion of the pons. The inferior portion of the lesion extended to the posterior and was surrounding the medulla, extending to the C1-C2 intervertebral level (Fig. 1a–e). A grossly subtotal surgical resection was done through the minimal lateral supraorbital approach (Fig. 1f–j). In the operation field, a shiny multi-lobulated cystic mass was seen on the cerebellopontine angle (Fig 2). The trigeminal nerve and seventh and eighth nerves were surrounded by the tumor. The cyst was filled with keratinous debris arranged in laminated layers. On histopathological examination, the cysts were thin-walled and lined by stratified squamous epithelium. Cystic contents included debris and keratin materials (Fig. 3). Based on these histopathological characteristics, the patient was diagnosed with an epidermoid cyst. The patient’s postoperative clinical course was uneventful, and neurological symptoms were improved. After the operation, the remnant cystic mass gradually decreased. In 15-month follow-up MR images, the remnant cyst mass completely disappeared (Fig. 1k–o).

Epidermoid tumors are rare congenital lesions originating from the ectoderm that constitute 0.3 to 1.8 % of all intracranial neoplasms [1, 2]. Intracranial epidermoid cysts are considered to arise from epithelial inclusions at the time of neural tube closure or during formation of the secondary cerebral vesicles, and have slow growth rates resembling that of the normal epidermis [1].

Pathologically, epidermoid cysts have well-circumscribed, irregular, thin walls with squamous epithelium lining. The epithelium undergoes progressive desquamation and keratin breakdown; therefore, the cystic contents include tissue debris, keratin, water, and solid cholesterol [2].

The typical imaging appearance is homogenous non-enhanced hypo-dense on CT, hypo-intense on T1-weighted MR imaging, and hyper-intense on T2-weighted and diffusion-weighted imaging. The CT density of the epidermoid cyst is usually between −2 and +10 Hounsfield units (HU) [3, 4]. These findings could be attributed to the lipid components and cholesterol. MR signals depend on the relative composition of cholesterol and keratin of the cystic contents. Generally, cholesterol in an epidermoid is in a solid state and appears hypo-intense on T1-weighted images. However, it occasionally presents as hyper-dense lesions on CT, making the diagnosis more difficult. Atypical intracranial epidermoid cysts show a hyper-dense signal on CT and hyper-intense signal on T1-weighted imaging as has been reported [5, 6, 8]. The suggested causes of the hyper-intensity on T1-weighted images include high protein concentration, mild calcification, and paramagnetic effects [8, 9]. According to Ahmadi et al., a protein level of 9.0 g or greater per 100 mL can increase the signal intensity of the cystic fluid on T1-weighted MR images [10]. Nagashima et al. also examined the total protein concentration of cystic fluid (15 g/dl) and suggested that the highly proteinaceous contents of the cyst contributed to the hyper-density [11]. The hyper-density could be attributed to the calcification of the keratinized debris and saponification of debris to calcium and also can be attributable to traumatic or spontaneous intracystic microbleeding, abundance of polymorphonuclear leukocytes, and deposition of ferrocalcium complex or iron-containing pigment [5, 6, 11, 12].

The treatment of choice for epidermoid cyst is a total resection of the tumor. But sometimes total resection is impossible because of anatomical complexity. This tumor was fortunately dissected easily from the adjacent structures. But we could not remove all of the tumor because of the long distance from the surgical field, not due to tumor adhesion. So some part of the tumor was left behind at the right upper cervical level. We periodically followed up the patient, and fortunately, the remnant lesion completely disappeared.

The 46-year-old man in our report suffered tingling sensation and numbness on both lower extremities. CT showed hyper-dense masses in both cerebellopontine areas which extended to the C1-C2 intervertebral level. The mass was hyper-intense on T1-weighted and hypo-intense on T2-weighted images.

The cerebellopontine angle is the most common site of the epidermoid tumors, followed by the prepontine, suprasellar, and parasellar cisterns in order. It is less frequently located in the parasellar region and the medial cranial fossa [1]. To the best of our knowledge, intracranial epidermoid tumors with extension into the upper cervical spinal canal are rare. There are only five such cases reported previously (Table 1). Of the five reported cases, only one had atypical hyper-intensity on T1-weighted images and hypo-intensity on T2-weighted images with upper cervical extension. The reason for the unusual downward extension into the upper cervical canal remains unclear. One possibility is that the cistern is anatomically narrower at the lower portion than at the upper portion of the posterior fossa, which would encourage inferior extension. Another possibility is that these tumors tend to spread in the direction of the main flow of the cerebrospinal fluid at the base of the posterior fossa [13].

**Table 1:** Reported intracranial epidermoid tumors with extension into the spinal canal

Author (year)	Age	Sex	Main location	Extension level	MR signal	Surgical removal
MacCarty et al. (1959) [14]	24	F	Rt. CP angle	Upper cervical	N/A	Subtotal
Keville and Wise (1959) [15]	62	Not reported	Fourth ventricle	Upper cervical	N/A	Partial
Ishii et al. (1983) [16]	53	M	Pontine	Upper cervical	N/A	Subtotal
Hasegawa et al. (1989) [13]	61	M	Rt. CP angle	C1-C2 intervertebral level	T2W SE: hyper-intensity	Subtotal
					IR: hypo-intensity	
Teo et al. (2006) [7]	27	F	Lt. CP angle	C2 level	T1W SE: hyper-intensity	Subtotal
					T2W SE: hypo-intensity	
Present case	46	M	Both CP angles	C1-C2 intervertebral level		Subtotal

## Conclusions

We report a case of epidermoid cyst that displayed hyper-intensity on T1-weighted MR images with upper cervical extension. We think the reason for the unusual downward extension into the upper cervical canal is related to the anatomical structure of the cistern and flow of the cerebrospinal fluid.

## Consent

Written informed consent was obtained from the patient for publication of this case report and any accompanying images. A copy of the written consent is available for review by the Editor-in-Chief of this journal.

## Abbreviations

CT: computed tomography
MR: magnetic resonance
